# Supplementary material for: An Inverted Perovskite Solar Cell with Good Comprehensive Performance Realized by Reducing the Concentration of Precursors
Source: Nanomaterials (Basel). 2022 May 19;12(10):1736. doi: 10.3390/nano12101736 (PMC9147920; doi:10.3390/nano12101736)
Supplement: Supplementary file 1 [file nanomaterials-12-01736-s001.zip › nanomaterials-1718465-supplementary.pdf]

Supplementary material

An Inverted Perovskite Solar Cell with Good Comprehensive Performance Realized by Reducing the Concentration of Precursors

Lijia Chen [1,*,†], Cunyun Xu [2,†], Yan Qin [3], Xiaofeng He [2], Hongyu Bian [2], Gaobo Xu [2], Lianbin Niu [1] and Qunliang Song [2,*]

1 College of Physics and Electronic Engineering, Chongqing Normal University, Chongqing 401331, China; niulb03@126.com
2 Institute for Clean Energy and Advanced Materials, School of Materials and Energy, Southwest University, Chongqing 400715, China; cyxuamos@foxmail.com (C.X.); hexf1992@163.com (X.H.); samuelhbian@163.com (H.B.); gbxu1805@163.com (G.X.)
3 Foundation Department, Army Logistics Academy, Chongqing 401331, China; qy.yfds@foxmail.com
* Correspondence: ljchen01@cqnu.edu.cn (L.C.); qlsong@swu.edu.cn (Q.S.)
† These authors contributed equally to this work.

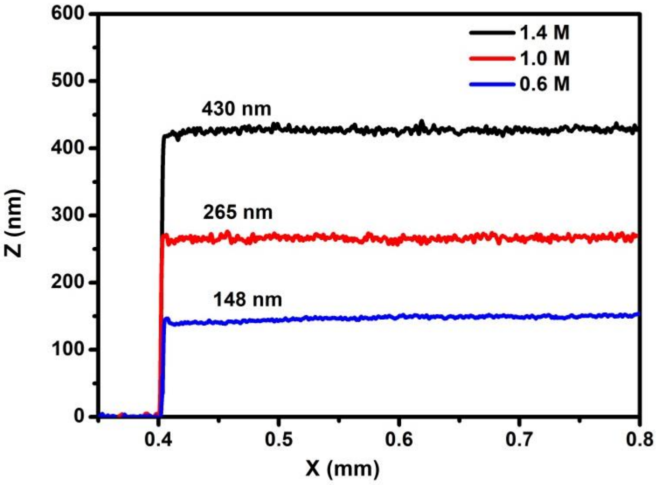

Figure S1. The thicknesses of perovskite films with different concentrations (1.4 M, 1.0 M and 0.6 M) of perovskite precursor. All the perovskite films were directly coated on ITO substrates.

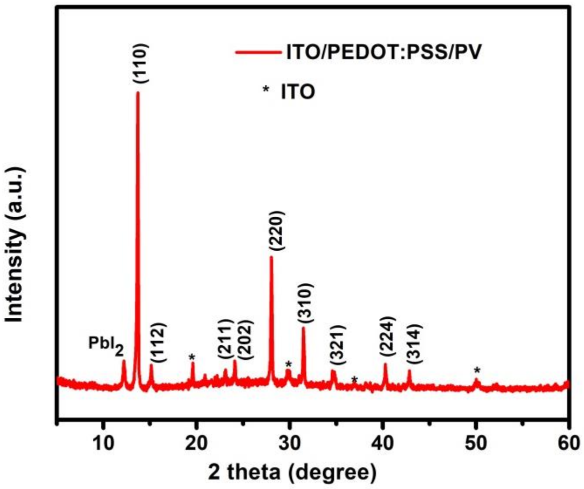

Figure S2. XRD spectra of perovskite film prepared by 1 M precursor coated on ITO/PEDOT:PSS film.

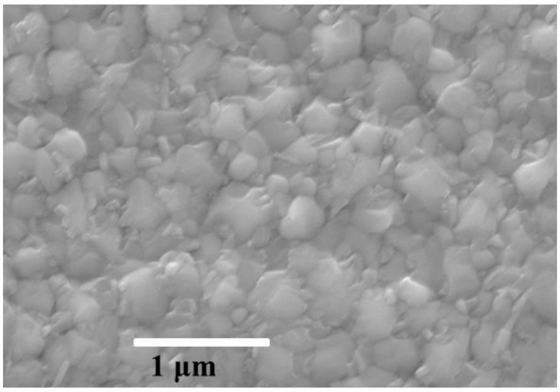

Figure S3: SEM image of perovskite film prepared by 1 M precursor coated on ITO/PEDOT:PSS substrate.

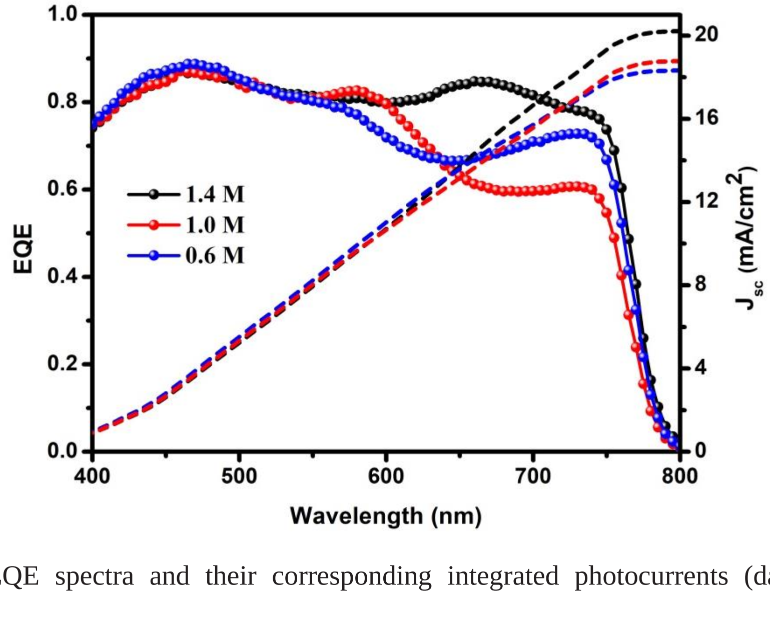

Figure S4. EQE spectra and their corresponding integrated photocurrents (dashed lines) of

inverted PSCs with different concentration (1.4 M, 1.0 M and 0.6 M) of perovskite precursors.

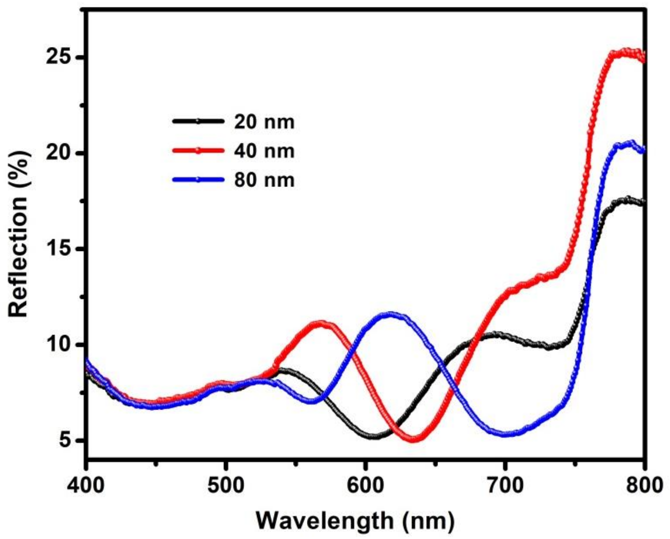

Figure S5. Reflection spectra of 1 M device with different thicknesses of C_{60} ETL.

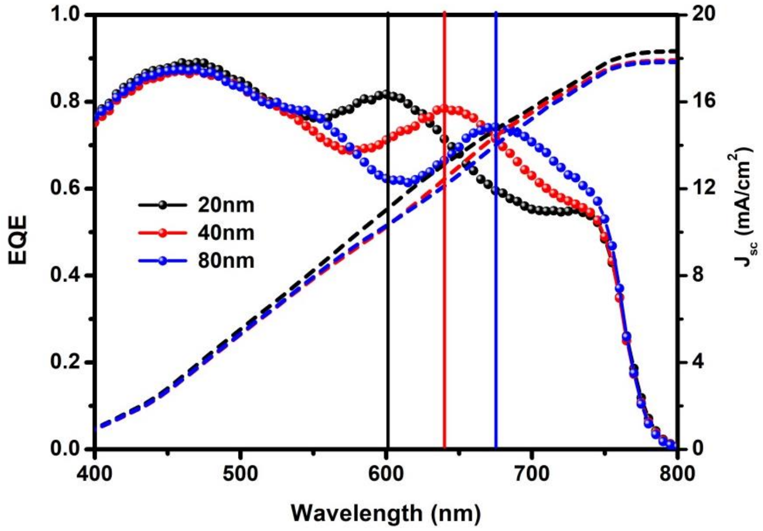

Figure S6. EQE spectra and their corresponding integrated photocurrents (dashed lines) of 1 M device with different thickness (20 nm, 40 nm, 80 nm) of C_{60} ETL.

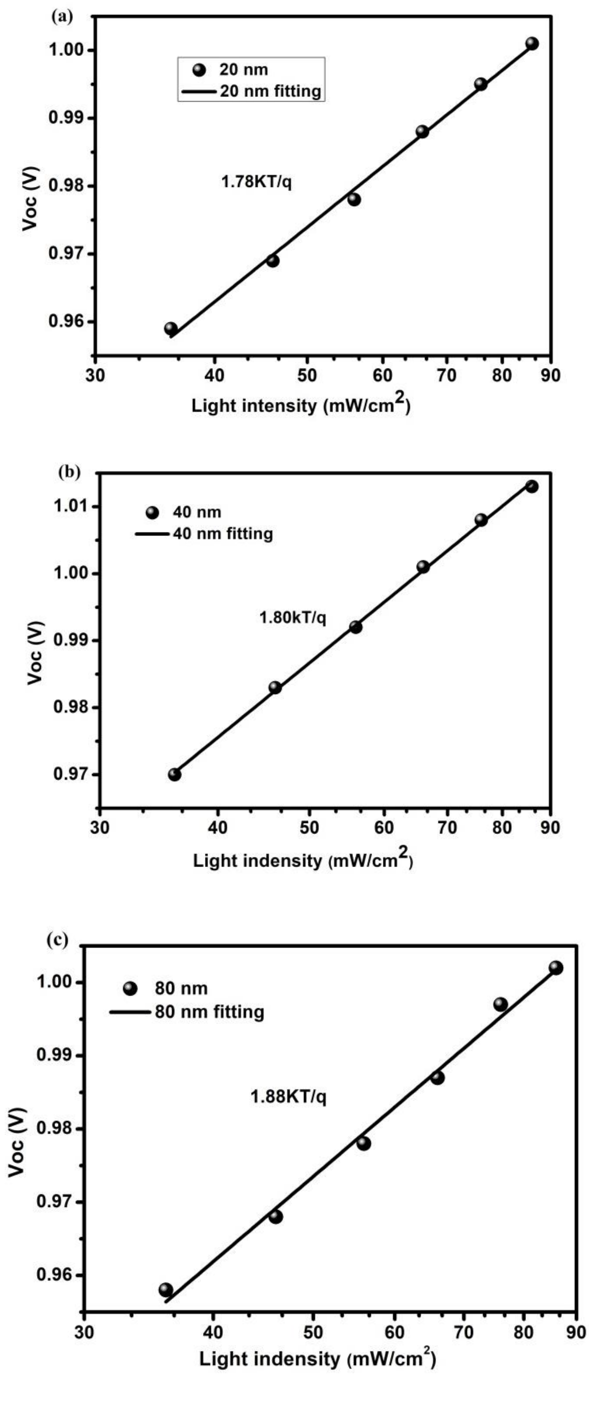

Figure S7. Voc *vs*. light intensity for the PSCs with diffirent thickness(a) 20 nm, (b)40 nm, (c) 80nm of C_{60} ETL

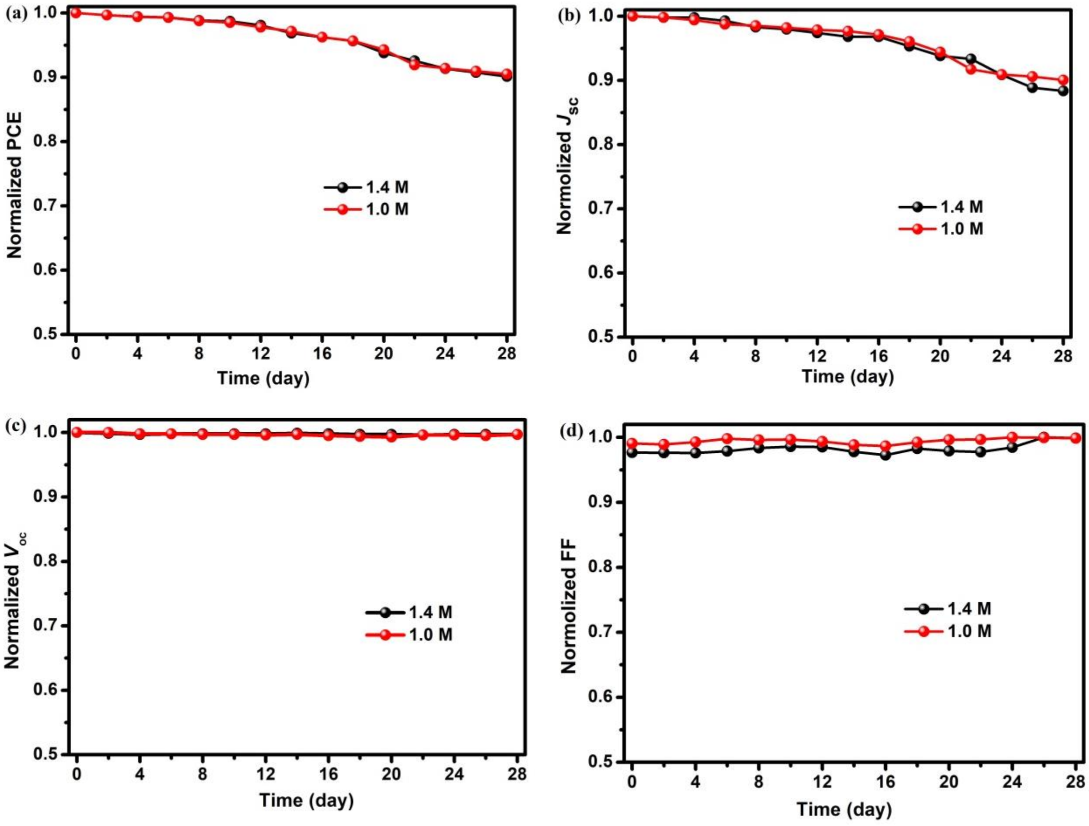

Figure S8. The normalized stability results displayed in Figure 4:(a) PCE, (b) J_{SC}, (c) V_{OC} and (d) FF.

Table S1: The photovoltaic parameters of 1 M device with three different thickness of C_{60} ETL.

Thickness C_{60}	V_{OC} **(V)**	J_{SC} **(mA/cm^2)**	***FF* (%)**	***PCE* (%)**
**20 nm**	1.030	18.53	81.2	15.50
**40 nm**	1.030	18.25	80.4	15.11
**80 nm**	1.028	17.30	80.6	14.33

Table S2: Parameters of equivalent circuit extracted from the fitting of impedance data for ITO/PEDOT:PSS/Perovskite/C_{60}/BCP/Ag device with different thicknesses of C60 (20, 40, 80 nm) under the illumination of AM 1.5G, 100 mW/cm^2.

Thickness C60	***R*s (Ω)**	***R*ct (Ω)**	***R*rec (Ω)**
**20 nm**	35.3	337	869
**40 nm**	35.8	352	373
**80 nm**	43.7	372	367
